# Zero-fluoroscopy pulmonary vein isolation with intracardiac echocardiography to monitor fetus in pregnant patient with atrial fibrillation

**DOI:** 10.1016/j.hrcr.2022.10.020

**Published:** 2022-11-09

**Authors:** Sharath C. Vipparthy, Jorge Gomez, Amit K. Mehrotra

**Affiliations:** ∗ Rush University Medical Centre, Chicago, Illinois; † University of Illinois College of Medicine at Peoria, Peoria, Illinois

**Keywords:** Pregnancy, Atrial fibrillation, Catheter ablation, Intracardiac echocardiography, Fetal monitoring, Zero-fluoroscopy

## Introduction

Atrial fibrillation (AF) is the most common arrhythmia in adults, affecting nearly 0.5%–1% of the general population and >8% of patients older than 80 years. However, it is rare among pregnant women, with an estimated incidence of 59.3 in 100,000 pregnancies.^1^ AF before pregnancy, mitral valve disease, congenital heart disease, and prepregnancy beta-blocker use were predictors of AF during pregnancy, with the highest incidence in the second trimester.^2^ AF is medically managed in pregnant women and pulmonary vein isolation (PVI) has not been reported in the literature. We report the first case of zero-fluoroscopy (zero-fluoro) PVI in a pregnant woman in her second trimester who failed medical therapy.

**Key Teaching Points**

• Atrial fibrillation (AF) is relatively rare in pregnancy and is mostly managed medically. Sotalol is preferred for rhythm control, whereas beta-blockers and digoxin are preferred for rate control. Cardioversion can be performed for hemodynamically unstable AF.
• Catheter ablation for AF has not been performed historically. Significant potential for maternal and fetal risks from electrophysiology study, anesthesia, and radiation exposure exists. However, in highly symptomatic patients zero-fluoroscopy catheter ablation can be considered.
• We describe a successful zero-fluoroscopy catheter ablation procedure in a pregnant woman in her second trimester who was highly symptomatic and failed sotalol therapy. We also describe the use of intracardiac echocardiography in monitoring the fetus during the procedure.

## Case report

A 36-year-old White woman, G7P4A2, in the 13th week of pregnancy presented to the office with 3–4 weeks of palpitations, anxiety, and chest pressure. The episodes were frequent and lasted up to 6 hours. Her physical exam was unremarkable, and body mass index was 26. She had frequent emergency room presentations for recurrent paroxysmal AF with rapid ventricular response. Eventually, sotalol was started at 80 mg twice daily. She was hospitalized within a week for recurrence of AF. Her medical history was significant for cavotricuspid isthmus line ablation for atrial flutter 3 years prior to presentation. Her father had AF at the age of 20 years. Laboratory results including blood counts, electrolytes, and thyroid stimulating hormone were in normal range. Echocardiogram showed no evidence of structural heart disease.

At our facility, sotalol dose was increased to 120 mg twice a day. Nevertheless, she continued to have recurrent episodes of AF. There was no evidence of QT prolongation on electrocardiogram or fetal bradycardia. She declined alternative antiarrhythmic therapy. After extensive informed discussion involving the anesthesia and high-risk obstetrics-gynecology departments, a decision was made to proceed with zero-fluoro PVI. At this time extensive discussion was held with anesthesia team and maternal fetal medicine colleagues and a decision was made to perform the procedure in the second trimester after completion of organogenesis, in case of a need for emergent fluoroscopy.

### Procedure details

**Figure 1:**
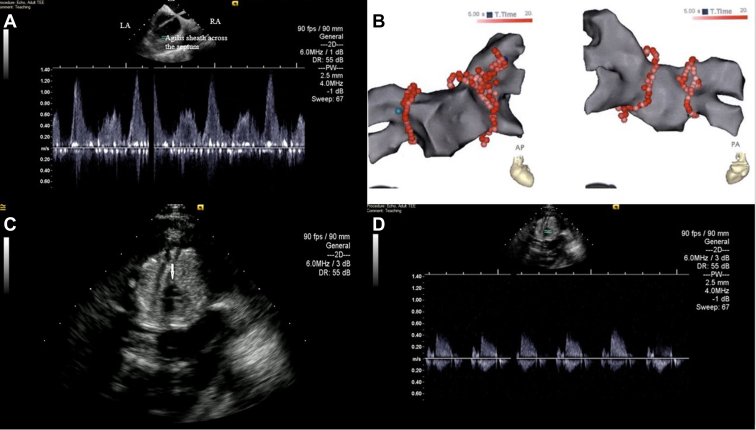
**A:** Intracardiac echocardiography (ICE) image of Agilis sheath (Abbott) just after transseptal puncture in the plane of left pulmonary veins as evidenced by pulmonary vein pulse vein Doppler images. LA = left atrium; RA = right atrium. **B:** Pulmonary vein isolation lesion set in anteroposterior (AP) (*left*) and posteroanterior (*right*) views; additional lesions were required to left superior pulmonary vein over the ridge to achieve complete isolation, as seen in AP view. **C:** ICE imaging of the fetus. From the initial view of the fetus in the long axis, clockwise rotation of the ICE probe revealed a short-axis view with the fetal cardiac atrioventricular (AV) valve (*white arrow*). **D:** Fetal AV valve Doppler. In the upper half of the image note the pulse wave Doppler cursor at the AV valve. In the lower half of the image note the sweep speed and Doppler for 3 seconds, estimating the fetal heart rate at 120 beats/min.

PVI was performed during the second trimester. The maternal-fetal medicine team assessed fetal heart rate (FHR) before and after ablation. Under general anesthesia and sterile precautions, standard venous access was obtained using ultrasound guidance. An ACUNAV (Siemens Healthcare, Barrington, IL) 8F intracardiac echocardiography (ICE) probe was used and demonstrated normal systolic function without pericardial effusion. A DECANAV (Biosense Webster Inc, Irvine, CA) catheter was placed in the coronary sinus and the CARTO 3 (Biosense Webster Inc, Irvine, CA) system used for mapping. Transseptal puncture was performed using a medium curl Agilis sheath (St. Jude Medical, St Paul, MN) and a Brockenbrough transseptal needle (Medtronic, Minneapolis, MN), with ICE guidance (Figure 1A). Heparin was administered to maintain an activated clotting time >350 seconds. A 5-spline multipolar PENTARAY (Biosense Webster Inc, Irvine, CA) mapping catheter was used for electroanatomic mapping. A ThermoCool SmartTouch SF radiofrequency (RF) ablation catheter (Biosense Webster Inc, Irvine, CA) was used for ablation.

The presenting rhythm was sinus. PVI was performed by wide-area circumferential ablation until entrance and exit block of all 4 pulmonary veins was achieved (Figure 1B). We performed continuous ablation with drag lesions, moving the catheter after 10–20 seconds, using 40 watts anteriorly, 20 watts posteriorly, targeting a force-time integral of 550 with 2 mm lesion size tagged on CARTO. An esophageal temperature probe was used to monitor temperature during posterior wall ablation. High-output pacing was performed on the anterior aspects of the right-sided veins to monitor for phrenic nerve injury. Procedure time was 2 hours 33 minutes, RF time 3232 seconds. A total of 30 RF lesions were administered. Extended ablation was required to the ridge for complete isolation of left superior pulmonary vein. Left atrial dwell time was 2 hours.

The procedure was well tolerated, without complications to the patient and fetus. The patient was hospitalized for 1 day for observation postprocedure. Enoxaparin was continued for 8 weeks after ablation.

Before, during, and after the case, fetal cardiac windows were obtained using the ICE catheter (Figure 1C, Supplementary Video 1). Fetal windows resembled atrioventricular (AV) valve views and a Doppler across the AV valve was obtained (Figure 1D). FHR was evaluated at the beginning and end of the case per maternal-fetal medicine recommendations, primarily calculated using external Doppler and stable around 120 beats per minute. FHR was also calculated using pulse wave Doppler across the fetal AV valves using the ICE catheter. The total time of general anesthesia was 4 hours and the anesthetic drugs used consisted of sevoflurane, propofol, and succinylcholine.

## Discussion

The treatment of arrhythmias during pregnancy is primarily medical. Medical therapy of AF in pregnant women is complicated owing to teratogenic potential, lack of significant safety data, higher risk of torsades de pointes, altered pharmacokinetics, etc.^3^

Rhythm control is the preferred strategy in pregnancy. Sotalol is considered safe in pregnancy.^4^^,^^5^ Amiodarone use is associated with neonatal hypothyroidism (17%–23%), delayed growth, and premature birth.^4^

ESC guidelines^4^ suggest the use of B1 selective beta-blockers (metoprolol or bisoprolol) for rate control, which are less likely to cause peripheral vasoconstriction, increased uterine contractility, and fetal growth retardation. Digoxin and verapamil are second line. Diltiazem is not preferred owing to the increased risk of fetal mortality, stillbirths, cleft palate, and skeletal, retinal, and heart malformations.

Cardioversion is recommended for hemodynamically unstable AF. Safety of cardioversion without maternal-fetal harm has been reported.^6^ Synchronized cardioversion using 50–100 J is effective in most cases.

Potential maternal and fetal risks from electrophysiology study and ablation include maternal tachycardia (induced and rapid pacing), anesthesia, and radiation exposure. Carcinogenesis, microcephaly, and mental retardation are primary concerns for radiation exposure, with thresholds for exposure reported to be 350–500 mGy and 500–1400 mGy for microcephaly and mental retardation, respectively.^7^

**Figure 2:**
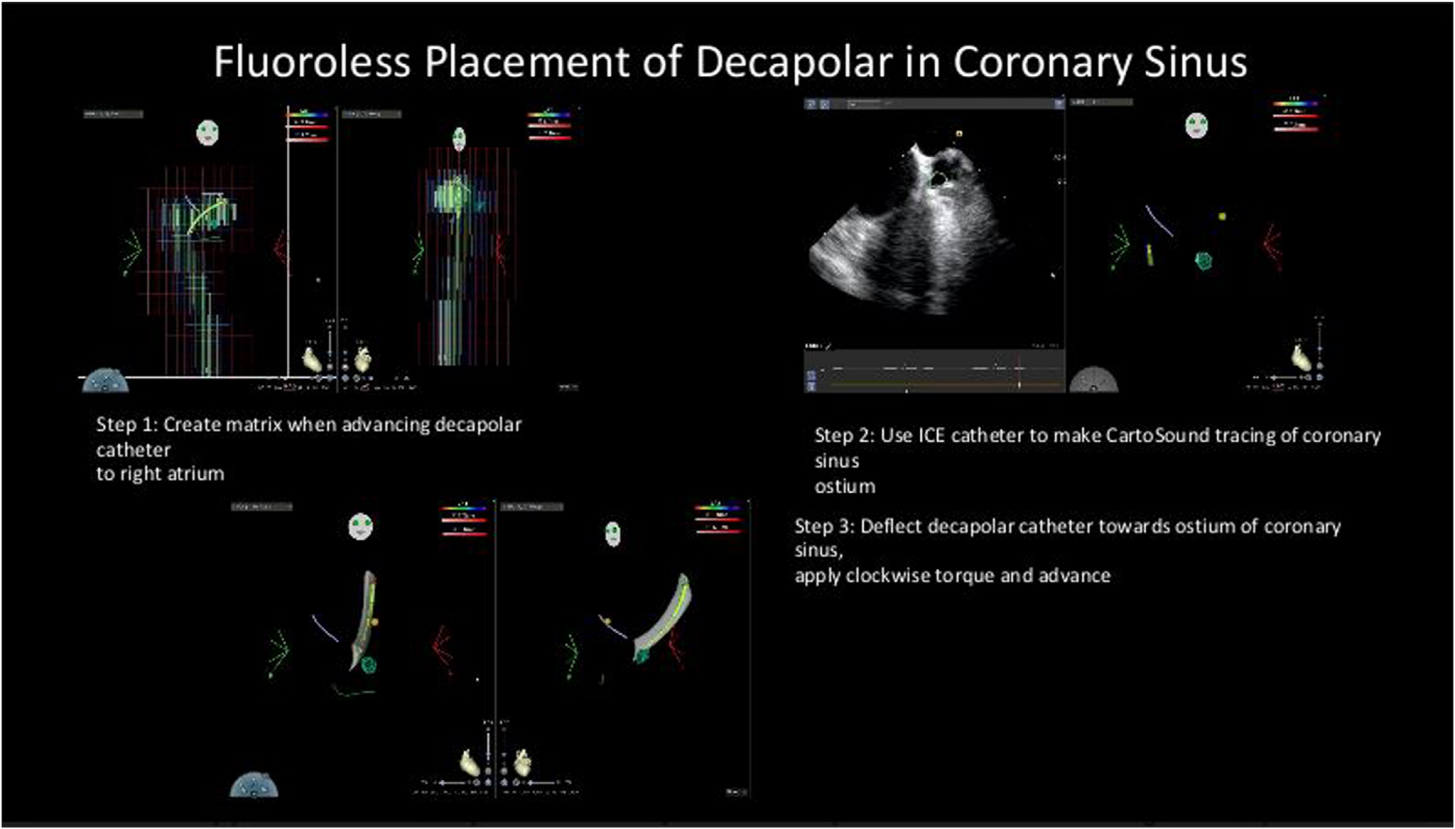
Zero-fluoroscopy DECANAV catheter (Biosense Webster) placement in coronary sinus (CS) (representative images obtained from a recent case). Step 1: Advancement of DECANAV catheter by feel, with simultaneous matrix and fast anatomic map creation and marking of His bundle. Step 2: CARTOSOUND mark-up of CS os. The right atrium / CS os are on the right side of screen and left atrium / mitral annulus are on the left side of screen on the intracardiac echocardiography (ICE) images. Step 3: Deflection of DECANAV catheter from His location to CS os and clockwise torque to advance into the CS.

**Figure 3:**
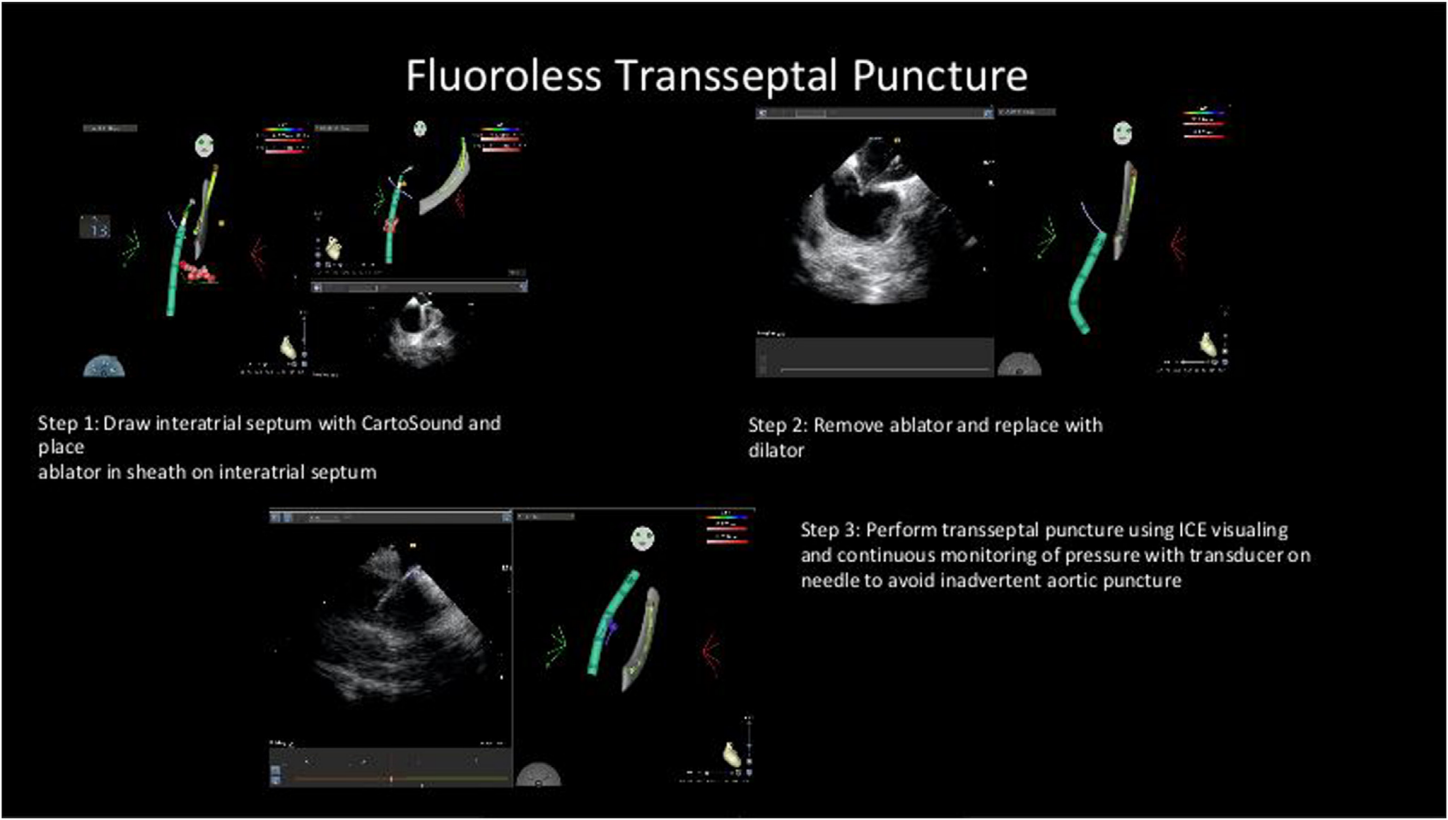
Zero-fluoroscopy transseptal puncture workflow. Representative images obtained from a recent case. Step 1: Atrial septum drawn on CARTOSOUND (Biosense Webster) at the plane of pulmonary veins and overlay imported to CARTO map. Step 2: Progressive advancement of ablation catheter and sheath to the septal line and visualizing the catheter and sheath position on intracardiac echocardiography (ICE). Once sheath is optimally positioned, exchange ablator for dilator and transseptal needle. Step 3: Transseptal puncture under direct ICE visualization and left atrium pressure monitoring.

Zero-fluoro ablation for drug-refractory premature ventricular contractions, supraventricular tachycardia, and ventricular tachycardia using EnSite NavX mapping system, CARTO mapping, and ICE have been reported.^7^^,^^8^ From our literature search, our case appears to be the first-ever reported zero-fluoro PVI for AF in a pregnant woman. Our patient chose to undergo ablation owing to recurrent symptomatic AF that did not respond to medical treatment. We used the CARTO mapping system along with ICE imaging as part of our workflow for zero-fluoro PVI ablation (Figures 2 and 3). Although we do not expect catheter ablation to be the mainstay of therapy for AF in pregnant women, we certainly hope that it remains an option for drug-refractory AF cases. Additionally, we are pleased to report the use of ICE in imaging the fetus during ablation. This was unique to our case and has never been reported. The ability of ICE to monitor FHR in the event of maternal hemodynamic instability during an electrophysiology study is an added advantage. FHR monitoring with ICE can be performed at the beginning, after left-side vein ablation and before switching to right-side vein ablation, during any hemodynamic disturbance (after ruling out maternal pericardial effusion), and at the end of the case.

Our patient was seen in clinic 8 weeks postprocedure. She was well and had no recurrence of AF despite discontinuation of antiarrhythmic drugs. She delivered a healthy newborn at 36 weeks of gestation. She elected no further follow-up appointments; however, a telephone follow-up at 3 years revealed that she continued to be symptom free.

## Conclusion

AF management during pregnancy can become challenging. Zero-fluoro ablation is a safe option when other approaches with rate-control agents and antiarrhythmic drugs have been exhausted without success at achieving both symptom and arrhythmia resolution.

## Appendix Supplementary Data

Supplemental Video 1 Title: ICE imaging of the Fetal Heart. Caption: From the initial view of the fetus in the long axis, clockwise rotation of the ICE probe revealed a short axis view with the fetal cardiac AV valve visible away from the probe.

## References

[1] Lee M.-S., Chen W., Zhang Z. (2016). Atrial fibrillation and atrial flutter in pregnant women - a population-based study. J Am Heart Assoc.

[2] Salam A.M., Ertekin E., van Hagen I.M. (2015). Atrial fibrillation or flutter during pregnancy in patients with structural heart disease, data from the ROPAC (Registry on Pregnancy and Cardiac Disease. JACC Clin Electrophysiol.

[3] Georgiopoulos G., Tsiachris D., Kordalis A. (2019). Pharmacotherapeutic strategies for atrial fibrillation in pregnancy. Expert Opin Pharmacother.

[4] Regitz-Zagrosek V., Roos-Hesselink J.W., Bauersachs J. (2018). 2018 ESC Guidelines for the management of cardiovascular diseases during pregnancy. Eur Heart J.

[5] Oudijk M.A., Ruskamp J.M., Ververs F.F.T. (2003). Treatment of fetal tachycardia with sotalol: transplacental pharmacokinetics and pharmacodynamics. J Am Coll Cardiol.

[6] Sengheiser C.J., Channer K.C. (2011). Recurrent atrial flutter and fibrillation in pregnancy. BMJ Case Rep.

[7] Driver K., Chisholm C.A., Darby A.E., Malhotra R., Dimarco J.P., Ferguson J.D. (2015). Catheter ablation of arrhythmia during pregnancy. J Cardiovasc Electrophysiol.

[8] Chen G., Sun G., Xu R. (2016). Zero-fluoroscopy catheter ablation of severe drug-resistant arrhythmia guided by Ensite NavX system during pregnancy: two case reports and literature review. Medicine (Baltimore).

